# ﻿*Typhoniummorangense* (Araceae), a new species from the tropical forest of Eastern Nepal

**DOI:** 10.3897/phytokeys.252.134081

**Published:** 2025-01-30

**Authors:** Rijan Ojha, Sudeep Rai, Harald Schneider

**Affiliations:** 1 Center for Integrative Conservation and Yunnan Key Laboratory for Conservation of Tropical Rainforests and Asian Elephants, Xishuangbanna Tropical Botanical Garden, Chinese Academy of Sciences, Mengla, Yunnan 666303, China Xishuangbanna Tropical Botanical Garden, Chinese Academy of Sciences Mengla China; 2 University of Chinese Academy of Sciences, Beijing, 100049, China University of Chinese Academy of Sciences Beijing China; 3 Biodiversity Research and Conservation Society, Kathmandu, Nepal Biodiversity Research and Conservation Society Kathmandu Nepal

**Keywords:** Bulbils, Koshi Province, Morang, *Typhoniuminopinatum*

## Abstract

A new species of the aroid genus *Typhonium* Schott has been discovered in Eastern Nepal. This species, named *Typhoniummorangense* R.Ojha & S.Rai, is described based on comparative morphological examinations with closely related species from the Indian subcontinent. The new species is currently known only from Pathari Shanishchare Municipality in the Morang district of Eastern Nepal. It is the second species of *Typhonium* observed in Nepal, and the only one endemic to the country. A detailed description, color plate, geographic distribution, phenology, and morphological comparison of the new species with morphologically close species are provided. Given its restricted range and the low number of individuals observed in the wild, this species requires special attention from conservation biologists working in Eastern Nepal.

## ﻿Introduction

The moderately species-rich aroid genus *Typhonium* Schott is expected to comprise around 100 species (Hetterscheid and Sookchaloem 2012), of which 72 species currently have accepted names, according to Plants of the World Online (POWO 2024). Phylogenetic studies have confirmed the placement of this genus in the tribe Areae, belonging to the subfamily Aroideae (Zhao et al. 2023). The genus is widely distributed across tropical to subtropical Asia, extending from the central Himalayas to New Guinea and Australia. More than half of the currently accepted *Typhonium* species are found in the Indochina region (Pham et al. 2023). Due to the high diversity and endemism of the genus in the Indochina region, it has long been considered the center of origin for the genus *Typhonium*. This is further supported by a recent biogeographic reconstruction study by Low et al. (2020), which suggests a middle to early Miocene origin (approximately 17.24 Ma) of the genus in the Indochina region. Within this region, the highest diversity (33 species) and endemism (23 species) are found in Thailand (Hetterscheid and Sookchaloem 2012; Sookchaloem and Maneeanakekul 2018). This center of biodiversity likely harbors several undiscovered species, as indicated by the recent discovery of *Typhoniumhangiae* V.D.Nguyen, D.D.Nguyen & V.C.Nguyen and *Typhoniumobtusum* Luu, X.B. Nguyen-Le & H.C. Nguyen in Vietnam (Pham et al. 2023; Luu et al. 2024). Given the known distribution of the genus, only a few species are found in the Himalayas. For example, only one species, *Typhoniumtrilobatum* (L.) Schott, has been reported in Nepal (Shrestha et al. 2022). As the northwestern distribution border of the genus, Nepal stands out as an important area for studying the diversity of *Typhonium*.

As part of our ongoing efforts to document the diversity of Araceae in Nepal, field surveys were conducted in the tropical forests of Eastern Nepal (Rajapaksha et al. 2023; Rai et al. 2024). In early 2024, a population of small aroids with 2–5 hastate to trilobate leaves and a pinkish-brown inflorescence was encountered. At first glance, these plants resembled the widespread *T.roxburghii* Schott or the more narrowly distributed *T.inopinatum* Prain, known to occur in India, Myanmar, and Thailand, due to the shape of their leaves, the inner color of the spathe, and the appearance of the spadix. However, detailed morphological examinations revealed these plants to be distinct from these two species and any previously described species. Thus, we introduce these plants as a new species endemic to Eastern Nepal.

## ﻿Materials and methods

To provide detailed information about the plant, we observed several individuals across different patchy stands. Mature individuals were collected to be deposited as herbarium specimens, while some inflorescences were preserved as pickled samples for further study. The morphometric study included a total of 20 individuals. Measurements of various parts of the living plants were taken in the field and photographs were captured using a Nikon Coolpix P900 camera. Micro-morphological measurements of flower parts of the specimens were examined using a dissection microscope. The images of these structures were taken using a mobile phone camera. Relevant literature was consulted to compare the morphology with closely related species and other species of the genus found in neighboring countries (Murata et al. 2010; Hetterscheid and Sookchaloem 2012; Venu and Rao 2014; Nirola and Das 2014; Manudev and Nampy 2022; Pham et al. 2023). Images of *Typhonium* specimens available in virtual herbaria and databases, including KATH (http://plantdatabase.kath.gov.np), CAL, ARUN, BM, P, PE (http://www.cvh.ac.cn), US, and others accessible through https://www.gbif.org and https://plants.jstor.org, were also examined (herbarium acronyms follow Thiers 2024). In addition to studying images of type specimens and protologs, the morphological characterization of putatively related species was based on descriptions provided by Li and Hetterscheid (2010) for *T.roxburghii*; King and Prain (1898), Venu and Rao (2014), and Luu et al. (2024) for *T.inopinatum*; Manudev and Nampy (2022) for *T.bulbiferum* Dalzell; and Murata et al. (2010) for *T.cordifolium* S.Y.Hu.

## ﻿Taxonomic treatment

### ﻿ Typhonium morangense

Taxon classificationPlantaeAlismatalesAraceae

R.Ojha & S.Rai sp. nov.

5D16B807-328C-5DD5-BAB3-0A90CF90306A

urn:lsid:ipni.org:names:77355953-1

Fig. 1

#### Type.

NEPAL, Koshi Province, Morang District, Pathari Shanishchare Munici­pality; 26°39'59"N, 87°33'29"E; ca.150 m; 2024.06.03; Sudeep Rai *MP011* [holotype KATH! isotypes KATH! [*MP012*], TUCH!, TURH!].

**Figure 1.**
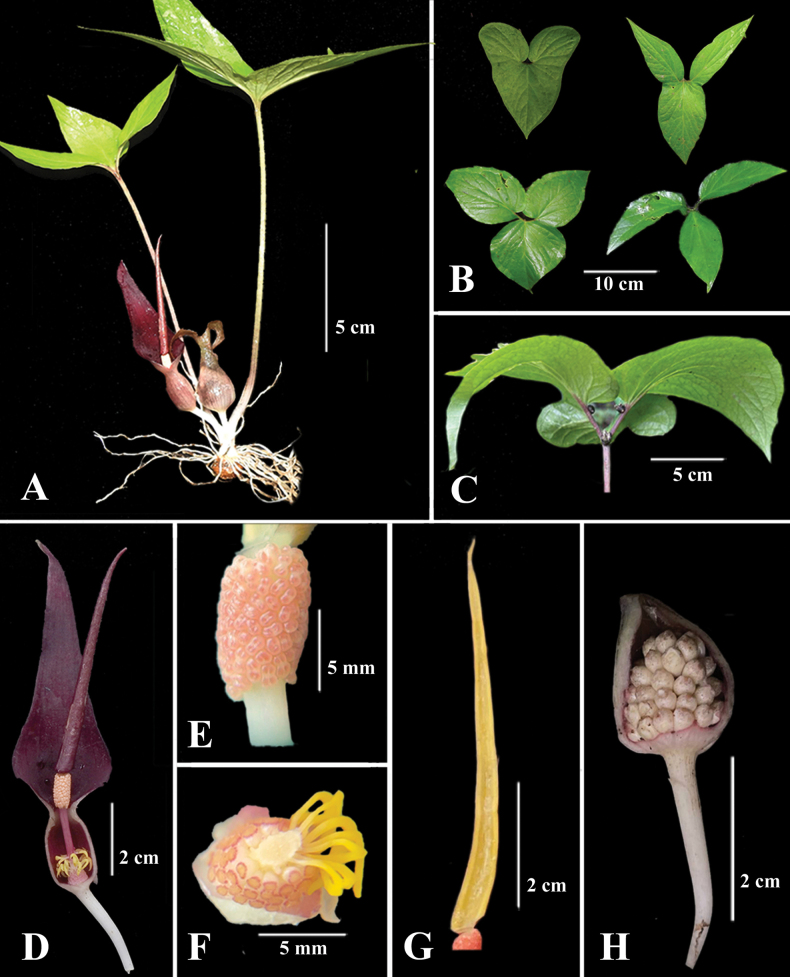
*Typhoniummorangense* sp. nov. **A** whole plant **B** different forms of leaf blade **C** bulbils **D** inflorescence **E** male zone **F** female zone and staminodes **G** longitudinal section of appendix **H** fruits.

#### Diagnosis.

*Typhoniummorangense* is morphologically closely related to *T.inopinatum* but it differs significantly from the latter species in having (1) smaller habit, 10–24 cm height (vs. 10–45 cm in *T.inopinatum*); (2) globose bulbils on top of petiole and leaves (vs. bulbils absent); (3) a sessile appendix (vs. subsessile); (4) thick, sickle-shaped staminodes (vs. filiform); (5) staminodes half curved downwards (vs. horizontally spread or, slightly curved); (6) ovary white (vs. yellowish) and (7) stigma pink (vs. yellow).

#### Description.

Seasonally dormant small herb, 10–24 cm tall; Tuber 0.8–2 cm long, 1–2 cm diameter, upright, sub-globose to sub-cylindrical with many filiform roots, without rhizomatous offsets. ***Leaves*** 2–5 together; ***petiole*** 10–23 cm long, light brown, grayish brown at base to light green at apex, globose black bulbil on the top of petiole. ***Leaf blade*** entire, hastate or shallowly or deeply tri-lobed or trifoliolate, glabrous, adaxially green, abaxially lighter green, 5–11 cm long, 2–7 cm wide when hastate, 10–12 cm when deeply trilobed, globose black bulbil on the basal margin of the leaf. ***Inflorescence*** solitary, usually 1–2 together, shorter than petiole; ***peduncle*** subterranean, white, 1.2–3.8 cm long; ***spathe*** 5.5–12 cm long, tube and limb separated by a constriction, grayish pink outside, inside dark reddish purple color; ***spathe tube*** ovoid to cylindric, 1–1.5 cm long, 1–1.7 cm diameter, outside grayish pink, inside reddish purple; ***spathe limb*** narrowly triangular, 4.5–10.5 cm long, 1–3 cm wide, smoothly tapering from below middle, apex acute; ***spadix*** 6.5–9.5 cm long, as long as spathe or slightly longer, sessile; ***female zone*** conical, 2–6 mm long, 4–5 mm diameter at the base, slightly pink, ***ovary*** unilocular, white with one dark yellow basal ovule, ***style*** absent, ***stigma*** sessile, disc shape with dark pink over the periphery; ***staminodes*** arranged in three whorls, free at the base, thick sickle-shaped, half-length curved downwards, yellow, glabrous, cover 1/3^th^ of the female zone; ***male zone*** 5–8 mm long, 3–5 mm diameter, cylindrical, ***stamens*** congested, ***thecae*** two, irregular, coral pink to sandy brown color with apical short slits or pores; ***appendix*** usually sessile, 2.7–7.7 cm long, narrowly elongated conical, dark reddish purple, base obliquely truncate, top acute, inside semisolid. ***Fruits*** ovoid to capsule-shaped, 0.2–0.3 cm diameter, 0.3–0.4 cm long, slightly light green at initial stages while turning white after maturity.

#### Phenology.

Flowering in May to June, fruiting July to August.

#### Etymology.

The specific epithet is based on the locality of its discovery, the Morang district of Eastern Nepal.

#### Distribution and habitat.

The new species is known from Pathari Shanishchare municipality, Morang district, growing under the canopy of dense *Shorearobusta* Gaertn. forest at ca. 150 m asl. This species prefers moist shady floors under the dense canopy of forest.

#### Uses.

No reports for utilization by the local human population are known.

#### Conservation status.

The new species has been recorded only from its type locality, where it forms patchy stands of approximately 150 individuals. However, it is highly likely to be present in similar nearby habitats. Together with the type locality, these undiscovered sites are expected to contain several hundreds of individuals. Until further investigation, the species is provisionally designated as “Data Deficient” (DD) following the IUCN standards (IUCN 2024). Conservation biologists active in Eastern Nepal may want to consider this species as requiring attention, as it is a local endemic known only from this region.

#### Taxonomic notes.

The hastate leaf shape, small inflorescence size, and presence of a few staminodes are shared characteristics between *Typhoniuminopinatum* and *T.morangense*. However, apart from key differences presented in diagnosis, *T.morangense* can be distinguished from *T.inopinatum* by the color of the spathe (grayish pink outside and reddish purple inside vs. basally brownish, apically green), the color of appendix (reddish purple vs. yellow, yellowish-brown), the length of the spadix (as long as or slightly longer than the spathe vs. shorter than the spathe), the female flowers (without style vs. with style). *T.morangense* also shares several similarities with *T.roxburghii*, in the habit and the color of spathe. However, *T.morangense* differs from *T.roxburghii* in several characteristics: the size of the inflorescence (spathe limb 4.5–10.5 × 1–3 cm vs. 13–15 × ~5 cm; appendix 2.7–7.7 cm vs. 12–15 cm), the arrangement of the staminodes (arranged in three whorls vs. more than three whorls; half-length curved downward vs. only the tips pointing downward), the appendix (sessile vs. stipitate), the shape of the ovule (ellipsoid vs. ovoid), and the color of the stigma (pink vs. purple). Although *T.inopinatum* and *T.roxburghii* share several similar characters with *T.morangense*, neither has bulbils.

Bulbils have also been reported in the Indian endemic *T.bulbiferum* and *T.cordifolium* distributed in Myanmar, Thailand, Vietnam, and Cambodia (Nguyen et al. 2022). These two species differ from *T.morangense* by having triangular to sagittate or cordate leaves in *T.bulbiferum* and ovate to elliptic leaves in *T.cordifolium* (vs. hastate, tri-lobed, or trifoliolate leaves in *T.morangense*). Additionally, *T.bulbiferum* has a linear-lanceolate, pale rose spathe with an acuminate apex, and *T.cordifolium* has a narrowly triangular-ovate, dark reddish-purple to purplish spathe with an acuminate and apically curled apex (vs. narrowly triangular, grayish-pink spathe with a dark reddish-purple interior and an acute apex in *T.morangense*).

It is noted that a plant from India with globose bulbils on the lower leaf margin and the petiole apex, which was identified as *T.roxburghii* by Nirola and Das (2014), was recently confirmed to be a misidentification (Manudev and Nampy 2022). This indicates uncertainty in identification due to the presence of bulbils.

A comprehensive morphological comparison of the new species with these related species is presented in Table 1.

**Table 1.** Morphological differences between *Typhoniummorangense* and closely related species.

	*T.morangense*	*T.bulbiferum*	*T.cordifolium*	*T.inopinatum*	*T.roxburghii*
**Height**	10–24 cm	15–30 cm	-	10–45 cm	10–35 cm
**Leaf blade**	hastate or tri-lobed or trifoliolate, entire, 5–11 × 2–12 cm	triangular hastate, sagittate or cordate, mid lobe 5–8 × 3–6 cm	narrowly ovate-elliptic to elliptic, 4–25 × 1.5–4 cm	ovate to triangular or hastate, entire, 8 × 2 cm long	hastate, triangular, shallowly or deeply tri-lobed, 5–17 × 4–14 cm
**Bulbils**	globose, black on the top of the petiole and leaves base	globose to linear bulbils	present on the apex of the leaf	absent	absent
**Spadix**	6.5–9.5 cm long, sessile, as long as spathe limb	slender, as long as spathe	as long as spathe	4.3–9 cm long, shorter than spathe limb	subequaling the spathe
**Appendix**	2.7–7.7 cm long, dark-reddish purple, usually sessile, base obliquely truncate, apex acute	7–14 cm long, pale yellow to cream, stipitate, rounded at the base	3.6–7.7 cm long, brick orange-colored, base not truncate	4–6 cm long, yellow, yellowish-brown, subsessile, base truncate	12–15 cm, narrowly conical, dark purple, truncate, pale red stipe
**Spathe tube**	ovoid to cylindrical, 1–1.5 cm long, 1–1.7 cm in diameter, outside grayish pink, inside reddish purple	oblong, 1–2 cm long.	1.2 × 1 cm long	ovoid, 2 cm cm long, 1.5 cm wide, greenish inside and outside	-
**Spathe limb**	narrowly triangular, 4.5–10.5 × 1–3 cm, grayish pink outside, dark reddish purple inside, acute apex	linear-lanceolate, 7–16 × 2–4 cm, hyaline or pale rose, acuminate at apex	narrowly triangular-ovate, 5–9 × 3 cm, dark reddish purple to purplish brown, apex acuminate, upright or recurved, and curled apically	narrowly ovate to lanceolate, 9–10 × 4.5 cm, greenish (light purple outside), green with dark purple streaks and spots inside, apex acute to acuminate	broadly triangular-ovate, 13–15 × ca. 5 cm, purple or purple mixed with a dirty green flush outside, inside deep rich purple, usually twisted apex
**Stamens**	coral pink to sandy brown	-	creamy white	pale yellow	yellow
**Staminod**	thick sickle-shaped, arranged in three whorls, half-length curved downwards, acute tip	acinaciformis, curved, uniseriate	cylindrical, spreading, obtuse tip	filiform, horizontally spread and slightly curved, bifurcated pointed or entire tip	filiform, weakly papillose, arranged in 3 whorls, horizontally spread, pointing downward, acute tips

For now, the new species is considered endemic to Nepal, but further research is necessary to confirm its distribution range, particularly the potential occurrence in India. Special attention should be given to critically studying specimens with globose bulbils.

## Supplementary Material

XML Treatment for Typhonium morangense

## ﻿Additional information

### Author ORCIDs

Rijan Ojha https://orcid.org/0000-0002-8270-6343

Sudeep Rai https://orcid.org/0009-0004-7697-9267

Harald Schneider https://orcid.org/0000-0002-4548-7268

### Data availability

All of the data that support the findings of this study are available in the main text.
